# Hepatitis B virus infection among different sex and age groups in Pakistani Punjab

**DOI:** 10.1186/1743-422X-8-225

**Published:** 2011-05-13

**Authors:** Fawad Khan, Sulaiman Shams, Ihteshamud Din Qureshi, Muhmmad Israr, Hayat Khan, Muhammad Tahir Sarwar, Muhammad Ilyas

**Affiliations:** 1 Test Care Diagnostic lab, Lahore, Pakistan; 2 National Centre of Excellence in Molecular Biology, University of the Punjab, Lahore; 3 NUST Center of Virology and Immunology (NCVI), National University of Sciences and Technology (NUST), Sector H-12, Kashmir Highway, Islamabad, Pakistan; 4 Fatima Jinnah Medical College Lahore, Pakistan

## Abstract

**Background:**

Hepatitis B virus (HBV) infection is a serious health problem in the developing countries including Pakistan. Various risk factors are responsible for the spread of this infectious disease. Prevalence of HBV infection in apparently suspected individual of Punjab province of Pakistan was analyzed during January 2008 to December 2010. Current study was aimed to investigate the epidemiology and risk factors of HBV infection.

**Methodology:**

Four thousand eight hundred and ninety patients suffering from chronic liver disease were screened for the presence of HBV DNA using qualitative Real Time PCR methodology to confirm their status of infection. A predesigned standard questionnaire was filled for all the patients that included information about the possible risk factors.

**Results:**

A total of 4890 ELISA positive patients were screened for Hepatitis B virus infection. Of these 3143 were positive for HBV, includes 68.15% males and 31.85% females. Male were observed to be more frequently infected as compared to the female with a positivity ratio of 2.14: 1. The rate of infection increases with the passage of time in the course of three years. Highest frequency of infection was found in the age of 21-30 was 34.93% followed by 23.83% in 31-40. Only (13.39%) were belonging to the age group 11-20 year. The rate of infection declines with increasing age as shown by age groups 41-50 (16.13%) and 51-60 (7.09%). While children aged 0-10 and very old >60 age groups were very less frequently 1.49% and 1.65% infected respectively. Important risk factors contributing to HBV spread include barber risk (23.60%), blood transfusion (4.04%), History of injection 26.19%, Reuse of syringes 26.60%, dental risk (11.20%) and surgical procedure (4.26%). Among the entire respondents trend sharing personal items was very common. History of injection, barber risk, surgery and dental procedure and reuse of syringes appear as major risk factors for the transmission.

**Conclusion:**

Male were more frequently exposed to the risk factors as compared to female. Similarly the younger age group had high rate of infection as compared to the children's and the older age groups. Reuse of syringes', barber risk and History of injection were main risk identified during the present study. To lower HBV transmission rate Government should take aggressive steps towards massive awareness and vaccination programs to decrease the burden of HBV from the Punjab province of Pakistan.

## Introduction

Hepatitis B previously known as serum hepatitis is an infectious liver disease caused by hepatitis B virus. HBV is a partially double-stranded circular DNA virus belongs to the hepadnaviridae family [1-3]. Hepatitis B virus infection is more communicable disease than HIV and HCV infection. It is 50 - 100 times more infectious than HIV and 10 times more infectious than hepatitis C virus. HBV is a silent killer disease of the liver with many carriers not realizing that they are infected with the virus. [4].

Nearly 2 billion people in the world are exposed to the virus, while 350 million of them are infected with chronic hepatitis B [5]. The prevalence of HBV is the highest among the developing countries of Asia, Africa and the Pacific Islands and the lowest among the developed countries of America, Western Europe and Australia. Pakistan being part of the developing world, has viral hepatitis is a major public health problem [6]. Studies indicated that the hepatitis B is a crucial public health problem in Pakistan with increased morbidity and mortality. According to WHO (World Health Organization), Pakistan falls under the endemic region with 3% HBV infected population [7-9]. Exposure rate of HBV in Pakistan is not known clearly but limited data shows 35-38% prevalence with 4% being carriers and 32% having anti-Hepatitis B virus surface antibodies through natural conversion [9].

Important factors contributing to HBV spread include unsafe use of therapeutic injections,[10] blood transfusion, [11] tattooing,[12] mother to child transmission [13] and unsafe sexual practices [14,15]. In Pakistan, therapeutic injections administered in health care settings have been identified as major and consistently reported risk factors for HBV. [16]

HBV can affect both male and female of different age groups. However, provincial level estimates in the most populous province of Pakistan regarding the epidemiology, risk factors and prevalence in different age and sex group for hepatitis B are currently not available. Therefore we initiated the current study with the purpose of finding out the prevalence and common risk factors among the male and female population of different age groups in this region of the country.

## Materials and methods

### 2.1. Blood Sample collection

A total of 4890 HBsAg ELISA positive blood samples from HBV carrier subjects were received at Test Care lab and Pathology Department Fatima Jinnah Medical College Lahore between 2008-2010 for the detection of HBV DNA by PCR. A blood sample of 3ml was collected in a vacutainer from each patient and the serum was separated to store at -20°C until processed. Standard protocol for reducing contamination was strictly followed.

### 2.2. ELISAs for HBsAg and HBeAg

All the patients were screened for HBsAg and HBeAg using 3^rd ^generation enzyme-linked Immunosorbant Assay (ELISA) (DRG Instruments, Germany) kits using the methodology described by the manufacturers.

### 2.3. HBV qualitative PCR and viral load

HBV qualitative PCR and viral load for ELISA HBV positive was done using SmartCycler II Real-time PCR (Cepheid, USA) using HBV DNA qualitative/quantitative kits (Sacace Biotechnologies, Italy) according to the kit protocol. Polymerase chain reaction (PCR) negative patients were excluded where as positive subjects were further selected for the study.

### 2.4. Statistical analysis

Data was analyzed and the summary statistic was carried out by a statistical package, SPSS version 16.0 for Windows. The results for all variables were given in the form of rates (%).

## Results

A total of 4890 ELISA HBV positive subject's samples received from different geographical locations of Punjab were screened for HBV infection. Table 1 shows demographic characteristics of the studied sample. Most of the responded were of low economic status. Out of these ELISA positive specimens 1747 were found negative by HBV Qualitative real time PCR and therefore were excluded from the study. Total of 3143 PCR positive for HBV infection that were further included in the study. Of these 2142 (68.15%) were male and 1001 (31.85%) were female. Male were found to be more frequently infected as compared to the female with a positivity ratio of 2.14: 1.0 respectively. The rate of infection in both male and female tends to increase with the passage of time. The highest rates of infection 44.54% were found in 2010 followed by 30.13% 2009 and 25.32% in 2008 respectively.

**Table 1:** Demographic and socioeconomic status of the patients

Sex		Marital status		
**Male**	Female	Single	Married	Divorced
**2142**	1001	1369	1720	53

**Literacy**		**Socioeconomic status**		

**Literate**	Illiterate	High	Middle lower	Lower
**1175**	1967	576	985	1581

**Figure 1:**
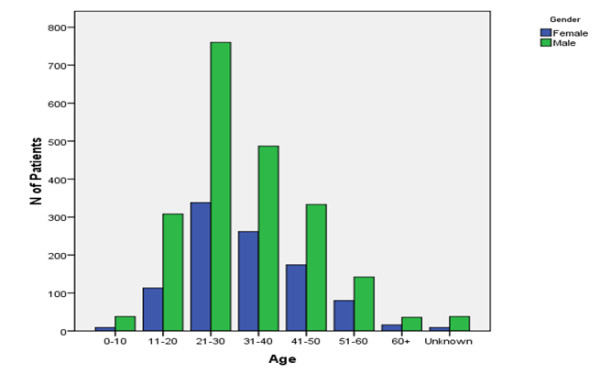
**The trend of HBV infection during the course of three years among the various age groups**.

Figure 1. shows the age wise distribution of hepatitis B virus infection in the current study. There was an age effect on the prevalence of hepatitis B infection in both genders. Highest frequency 34.93% of HBsAg positive individuals were found in the youngest age group with more positive individuals falling under 21-30 age group while 31-40 being the second 23.83% most abundant. 421(13.39%) individuals positive for HBV infection were belonging to the age group 11-20 year. The rate of infection declines with increasing age as shown by age groups 41-50 (16.13%) and 51-60 (7.09%) in the figure. While very young 0-10 and very old >60 age groups were very less frequently 1.49% and 1.65% infected by Hepatitis B virus. Individuals whose age is unknown have very low rate 1.49% of infection as shows in Figure 1.

Patients were randomly interviewed for the various risk factors to find out the possible modes of transmission. Table 2. shows the results of risk factors analysis for HBV infection in the studied population. The risk factors distributions were shaving from community barber, dental procedures, past surgical history, blood transfusion, Ruse of syringes and history of injection. Among the overall HBV DNA positive cases there was a high trend of sharing personal things. The study revealed that the three main risk factors identified were a History of injection 26.19%, reuse of syringes 26.60% and barber risk was 23.60%. There was also a strong 11.20% and 4.26% relation with the antecedent of dental, surgical procedures and 4.04% of Blood Transfusion. There was a lack of precision regarding any surgical procedure.

**Table 2:** Risk factors for hepatitis B virus infection in the respondents.

Risk Factors	Total	Observed %
**Blood Transfusion**	127	4.04%
**Barber risk**	728	23.16%
**Surgical procedure**	134	4.26%
**Dental risk**	352	11.20%
**Reuse of syringes**	836	26.60%
**Sharing personal item**	142	4.51%
**History of injection**	823	26.19%

More than one category per subject is possible

## Discussion

HBV infection is a global health problem with its continuously increasing burden on the developing countries like Pakistan [17]. No study on HBV epidemiology and pattern of transmission representing all the geographical regions of Punjab is available. The current study was initiated with the purpose of finding out the prevalence and the common risk factors among the different age groups of the studied region. Table 1 shows demographic distribution of all the respondents. This study revealed several high risk behaviors and practices for the transmission of this infection are significantly more prevalent among these patients as compared to the normal population. During the study we found that 64.27% infected population out of the study specimens. Because almost all the individuals were clinically infected patients who were referred to the hospitals and medical centers for their diagnosis and medication, this figure however dose not represents the overall picture of the total country population prevalence rate infected with hepatitis B virus. There are no recent national reports on the prevalence of hepatitis B infection in Punjab. However, studies performed in some selected populations may still be suitable for comparative purposes. Alam *et al*. 2007; reported a positivity ratio of 2.23: 1 [17] for male to female ratio with is quite matching with our value of positivity 2.14: 1.

Regarding the sex distribution of HBV infection there were more male 68.15% patients than female 31.15%. This was compatible with work form Naz *et al *2002 reported a high prevalence in males 68.3% than females 31.7% [18] which is quite comparable with our results. Mansoor *et al *2007 reported a high prevalence 64% in males than 36% females [19]. Moosa *et al*. 2009; and Awan *et al*. 2010; reported a high (59.1%, 58.3%) prevalence in males than females (40.9%, 41.7%) respectively [20,21]. Zubair *et al*.2010; determining the frequency of hepatitis B virus among children with chronic liver disease also find out a high 54% prevalence in male than females 46% [22]. Nwokediuko 2010; also reported a significantly higher (79.2%) infection rate in male as compared to the female (20.8%) [23]. Higher HBV Infection in males as compared to female may be due their being employed outsides their homes, visiting barber shops and also their involvement in blood transfusion practices. While women are mostly involved in house hold activates based o the social, cultural and religious preferences and influence.

Prevalence data from individual studies were further segregated into age groups. There was an age effect on the prevalence of hepatitis B infection. Prevalence rose from 13.39% in teenage 11-20 to a peak of 34.93% and 23.83 % in people aged 21-30 and 31-40 years respectively. After this it declined to 16.13% and 7.09% in people aged 41-50 and >6o years. While very young 0-10 and very old >60 age groups were very less frequently 1.49% and 1.65% infected by Hepatitis B virus infection. Age related prevalence for those having unknown age was 1.49% of infection. Alam *et al*. 2007; also reported a significantly higher infection in persons with age between 21-40 years followed by 41-60 years age [17]. Very young and old individuals were very less frequently infected by HBV. Castolo *et al*. 2001; report also supported our finding that prevalence of HBV infection is higher in patients up to the age of 40 years [24]. HBV infection being higher in young's respondents may be due to their greater exposures and interaction in society as compared to children and aged persons.

Unnecessary injections were given commonly among the respondents. History of therapeutic injection use for various ailments was present in over 26.19% patients suffering from hepatitis B virus infection. According to Ali *et al*. 2009; an average person receives four injections per year, most of which are unnecessary and up to 75% are unsafe or reused [25]. Also 26.60% respondents have the history of the syringe reuse. Syringes are reused and sterility of injections is often not maintained due to financial constraints. Qureshi *et al*. 2009; also reported intramuscular injection as a source of HBV and HCV [26]. Alavian *et al*. 2007; and Ali *et al*. 2009; identified reused syringes as a major risk factor for hepatitis B and C infection. Injections appear to be the single most significant factor in the spread of HBV and HCV in the general population of Pakistan [25,27]. This may be due to the lack of awareness about the possible risk factors among the healthcare providers and the population in general. Most of the patients were of the view that injected medicines are more effective than oral medications.

Shaving by barbers according to Waheed *et al*. 2010; in countries like Bangladesh, Pakistan, India, Iran, Israel and Italy, indicated that HBV can be transferred by blade sharing and barber related instruments [28]. During the study most 23.60% of the respondents were unaware that contaminated razors may also be one of the main causative sources for the transmission of hepatitis B virus infection. These results were supported by the findings of Janjua and Nizamy, 1999; in a cross sectional study of barbers in Rawalpindi/Islamabad showed barbers having very weak or no sterilization practices [29]. Moosa *et al*. 2009; and Qureshi *et al*. 2009; also reported that shaving by barber as the most frequent risk of getting HBV infection [20,26]. This may be due to the lack of awareness about risk factors among the barber community, reuse of blade and poor sterilization practices.

During our study 4.04% of the respondents had the history of blood transfusion. Patients with prior history are more prone to the infection as reported in the literature. Alavian 2007; as blood significantly contributes to the transmission of HBV infection [27]. Castolo *et al*. 2001; *and *Ali *et al*. 2009; reported a high rate of HBV infection in multi-transfused persons [24,25]. Qureshi *et al*. 2009; also reported blood transfusion as a potential risk of getting HCV and in HBV infection [26]. A high risk of HBV infection in most of the third world countries like Pakistan is due to lack of proper screening of blood.

According to our findings patient with prior history of dental treatment were 11.20% among the respondents. Our finding coincide the results of Castolo *et al*. 2001; that HBV is 2-fold higher in patients getting dental treatment [24]. Similarly Qureshi *et al*. 2009; and Moosa *et al*. 2009; also reported a higher (36%) and 41.3% patients infected with HBV after following dental procedure [20,26].

Patients with any kind of surgical history were 4.26% of the total respondents. Qureshi *et al*. 2009; reported history of Past surgery was present in 23% of HCV and 15% of HBV [26]. In 2009 Moosa *et al *also demonstrated surgical procedure as a risk of getting HBV and HVC infection among 28.8% of respondents [20]. Shaving by barbers, blood transfusion, surgery and dental treatments was also recognized by Ali *et al*. 2009; as a potential source of HBV transmission in Pakistan [25]. The reason might include lack of poor sterilization techniques and reuse of contaminated dental and surgical equipment.

## Conclusion and Recommendations

This study reveals the gender-wise prevalence and risk factors associated with HBV among the different age groups in the Punjab province of Pakistan. Blood transfusion, surgery and dental treatment appear as major risk factors for the transmission. Male were more frequently exposed to the risk factors as compared to female. Similarly the younger age group have high rate of infection as compared to the children's and the older age groups. This high prevalence was due to the lack of awareness regarding various risk factors involved in HBV transmission among most of the respondents. These risks are minimized by comprehensive measures in both public and private sector to ensure the need for regulation and control of the transfusion practices in Pakistan. In order to prevent HBV infection in our country government should take aggressive steps towards awareness programs involving both the media and public sectors organizations. Information should also be provided to the public that hepatitis B is vaccine preventable disease. Massive awareness and vaccination programs are required to decrease the future burden of HBV from Pakistani population.

## Abbreviations

HBV: hepatitis B virus; HCV: hepatitis B virus; DNA: Deoxyribonucleic acid; PCR Polymerase chain reaction; SPSS Statistical package software system; HBsAg: Hepatitis B surface antigen; HBeAg Hepatitis B e antigen.

## Competing interests

The authors declare that they have no competing interests.

## Authors' contributions

FK conceived the study, participated in its design, coordination and gave a critical view of manuscript writing. SS, HK and IDQ collected epidemiological data. MI and MI analyzed the data statistically. MTS and HK helped FK in giving critical view of manuscript writing and participated in statistical data analysis. All the authors read and approved the final manuscript.
